# Supplementary material for: Transition metal dichalcogenide metamaterials with atomic precision
Source: Nat Commun. 2020 Sep 14;11:4604. doi: 10.1038/s41467-020-18428-2 (PMC7490684; doi:10.1038/s41467-020-18428-2)
Supplement: Supplementary file 1 — Supplementary Information [file 41467_2020_18428_MOESM1_ESM.pdf]

Supplementary Information for

Transition metal dichalcogenide metamaterials with atomic precision

Battulga Munkhbat[1], Andrew B. Yankovich[1], Denis G. Baranov[1,2], Ruggero Verre[1], Eva Olsson[1], and Timur O. Shegai[1,]*

Supplementary Notes

a. Etching rate:

The etching rate was extracted by following the time evolution of circular holes along two principal directions - corner and side (Supplementary Fig. 2). Supplementary Figure 2b shows the cumulative thickness of the etched material as a function of etching time in the case of 1 µm radius hole for a (red) zigzag and (blue) armchair edges. The etching of the zigzag edges is rather slow, whereas for the armchair edge it is faster and more aggressive within first 8 min. After 8 min the etching rate slows considerably for both the zigzag and armchair edges due to the prevalence of the more stable zigzag edges. The average etching rates within the first 8 min for zigzag and armchair planes are 2.8 nm/min and 21.7 nm/min, respectively, revealing this process exhibits an etch rate selectivity of ~10 times faster for the armchair edges compared to than zigzag edges. However, the etching rates are not constant and typically slow down, especially for the armchair edges because of the gradual change from armchair to zigzag edges during the etching process.

b. TEM study of edges:

TEM images also reveal that on top of the etched zigzag surfaces a thin layer (0-3 nm) of amorphous material occasionally appears (Fig. 1e and Supplementary Figure 4). This material is likely left-over residues originating either from the etching process or more likely from the scotch-tape and/or PDMS stamps used during the dry-transfer process for TEM sample preparation after the etching process (see Methods). Supplementary Figures 7-8 show EELS spectrum image data from an etched flake, revealing the spatial variations of the major elements present in the sample. The EELS core-loss signal maps using the W O and S L edges (Supplementary Figure 7b-c and Supplementary Figure 8b-c) show both W and S exhibit very sharp signals at the etched edges, especially at the hexagon corners, consistent with faceted zigzag edge surfaces. In addition, there are also weak signals coming from O, Si, and C in the EELS spectrum image data. The O K and Si L signal maps appear to be correlated, with both exhibiting stronger signals at the etched surfaces (Supplementary Figure 7d-e and Supplementary Figure 8d-e). A line profile across the etched surface show both Si and O signals are peaked at the etched surface and have a layer thickness of a few nm. In addition, the Si L edge shape is consistent with spectra from SiO_2, but not Si, SiC, or SiN (Supplementary Figure 8h), implying the Si is bonded to O. The C L signal map shows that C is randomly located on all surfaces, with higher concentrations often found at the hexagon corners (Supplementary

Figures 7f and 8f). The source of the Si, O, and C signals are unknown, but they are likely to be residual material from the etching or PDMS stamping processes.

c. Additional structures:

Bulls' eye:

We have verified that it is possible to pattern more complex single hole shapes, such as bull's eye, using this wet anisotropic etching method. To illustrate effect of the wet etching on the inner and outer edges, generic bulls-eye and doughnut structures were fabricated that produce unexpected patterns in TMD flake (Supplementary Figure 10). Interestingly, the inner edges of the etched bulls-eye structures have well-defined zigzag edges, while the outer edges are rotated by 30^0 and have less-distinct armchair character. This indicates that donut and bulls-eye structures contain alternating metallic zigzag inner edges and semiconducting armchair outer edges. Therefore, either exclusively zigzag or armchair edges can be engineered with the anisotropic etching by controlling the initial structures.

Other TMDs:

In addition to WS_2 multilayers, the method has been successfully applied to anisotropically etch other TMD materials, such as MoS_2, $MoSe_2$, and WSe_2 (Supplementary Figure 11), as well as stacked heterostructures (Fig. 2c and Supplementary Figure 12). In all such cases the wet etching process created hexagonal holes. These studies reveal that the etching rate is different for different TMD materials and increases in the order: $MoS_2 < WS_2 < MoSe_2 < WSe_2$. Interestingly, the hexagonal holes etched into Mo-based TMDs have slightly tilted side edges with respect to [0001], whereas WS_2 yields near-vertical side edges. In addition to the high quality MoS_2 flake (HQ-graphene), the anisotropic etching was successfully tested on MoS_2 flakes exfoliated from a mineral rock (Supplementary Figure 11b).

d. Etching monolayers:

The monolayer of WS_2 withstands the wet etching process as shown in the PL image (Supplementary Figure 13a). However, the circular holes in the monolayer cannot be directly converted into hexagonal shapes. Instead, the resulting holes exhibit saw-teeth like edges (Supplementary Figure 13a). This can be due to several reasons, for instance, substrate roughness or direct contact of the monolayer with the substrate as well as different chemical reactivity of monolayer compared to multilayer. To get further insight into anisotropic etching of monolayer, we performed additional experiments and the key results are summarized in Supplementary Figure 13b-e. In order to provide a smoother substrate as well as to prevent direct contact with the substrate, we prepared several heterostructure samples by transferring a WS_2 monolayer on top of another pre-transferred WS_2 monolayer on Si/SiO_2 substrate. First, the so obtained WS_2 monolayer heterostructures are patterned with circular 500 nm holes. Subsequently, the pre-patterned monolayer samples were wet-etched with the standard etchant,

but at elevated temperature of ~80^oC, to speed up the etching process. As can be seen in Fig. S13b, the initial circular hole in monolayer WS_2 is transformed into an irregular pattern with pronounced saw-teeth edges after 7 min of etching. By extending the etching to 12 min, a more-pronounced triangular hole is obtained (Supplementary Figure 13c). As the etching process and size of initial circular holes can be controlled, we managed to obtain triangular holes in WS_2 monolayer starting with smaller initial holes (Supplementary Figure 13d-e). We also prepared a heterostructure that consists of a monolayer on top of multilayer WS_2 flake on Si/SiO_2 substrate and performed above-mentioned fabrication processes to do anisotropic wet etching. However, it was extremely difficult to draw conclusion and we observed anisotropically etched only hexagonal holes in the multilayer, since the multilayer underneath is anisotropically etched much faster than top monolayer.

Supplementary Figures:

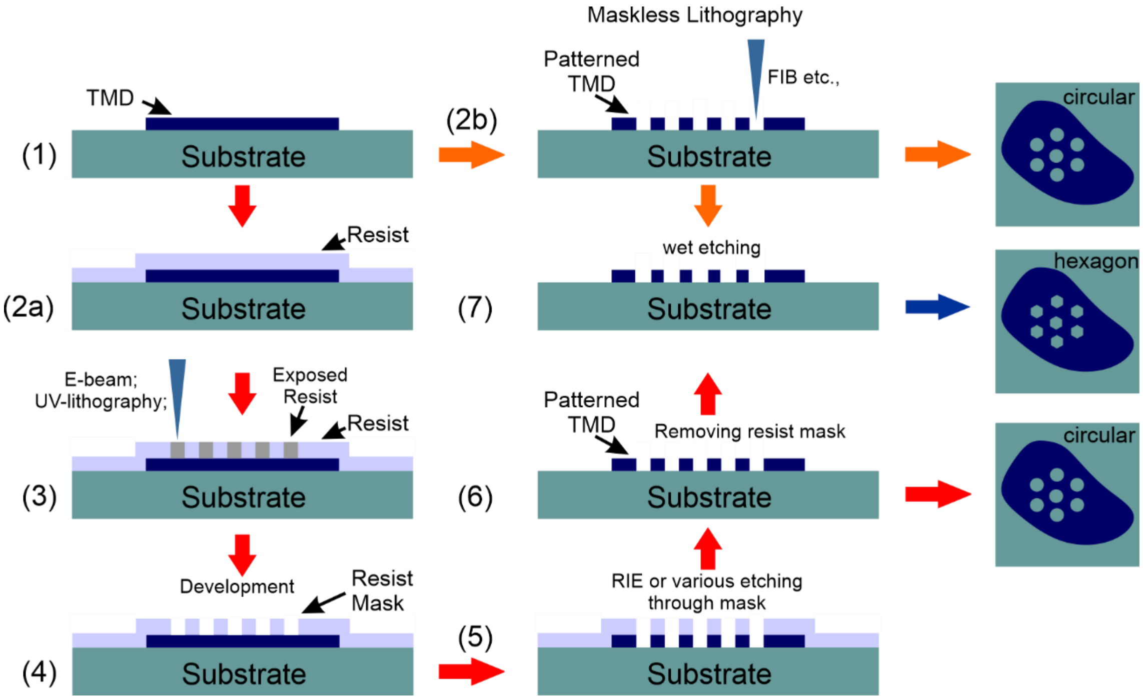

Supplementary Figure 1. Scheme for the fabrication method. (1) Transferring of mechanically-exfoliated TMD onto a substrate. (2a) Spin coating of resist for either e-beam, laser writing, or UV-lithography. (2b) Focused-ion beam milling to create an initial pattern for further anisotropic etching. (3) Exposing the resist to create a mask with various lithography techniques. (4) Development of the resist. (5) Etching TMD through the mask to create an initial pattern for further anisotropic etching. (6) Removal of the remaining resist. (7) Anisotropic wet-etching resulting in hexagonal holes.

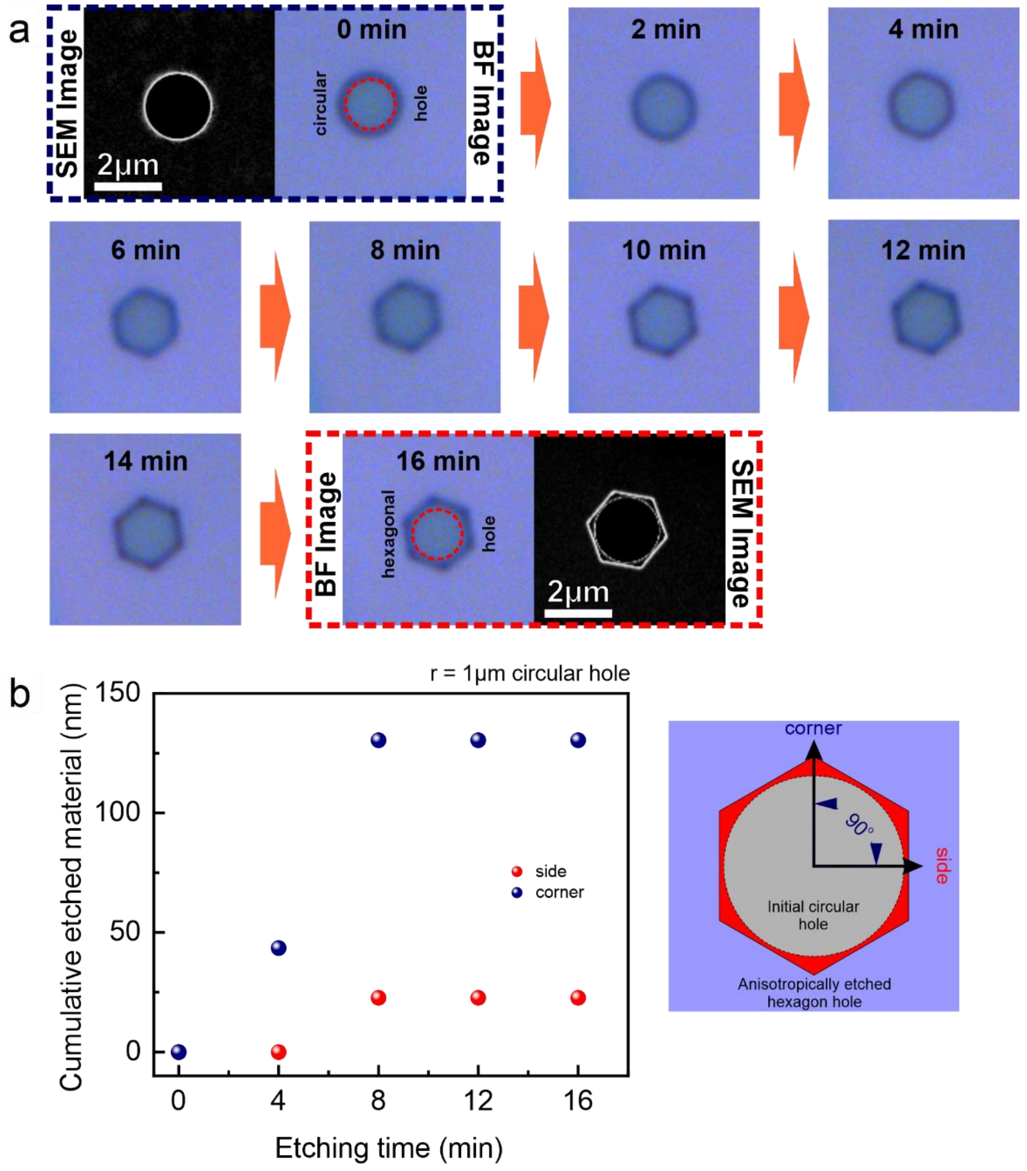

Supplementary Figure 2. (a) Time evolution of the anisotropic etching process starting with a single circular hole using the H_2O_2:NH_4OH:H_2O etching solution at 50 ºC. The two black and white images are SEM images before and after 16 min of anisotropic etching. The colored images are a series of bright field image taken every 2 min of anisotropic etching after being rinsed with water and dried with nitrogen. (b) Plot of the cumulative amount of etched material as a function of etching time for a 1μm radius hole in the directions along the side (red-dots: along the zigzag plane) and corner (blue-dots: along the armchair plane) of the hexagonal hole. The average etching rate for zigzag and armchair in the first 8 min, are 2.8 nm/min and 21.7 nm/min, respectively, revealing the armchair planes have an etch rate selectivity of ~10 times larger than the zigzag planes under above-mentioned condition.

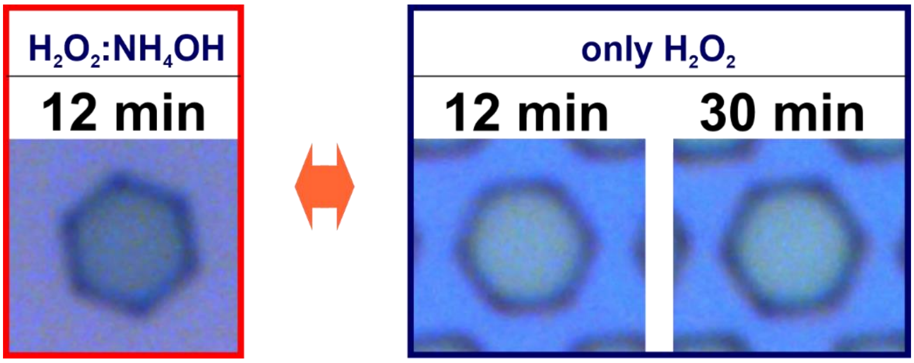

Supplementary Figure 3. Comparison between the etching process with and without NH_4OH. An initial circular hole is converted into hexagonal hole within 12 min using the standard etching solution composed of H_2O_2:NH_4OH:H_2O (1:1:10), whereas using an etchant composed of only H_2O_2:H_2O (1:10) at 50 ºC, it takes a longer time to be fully converted into a hexagonal hole.

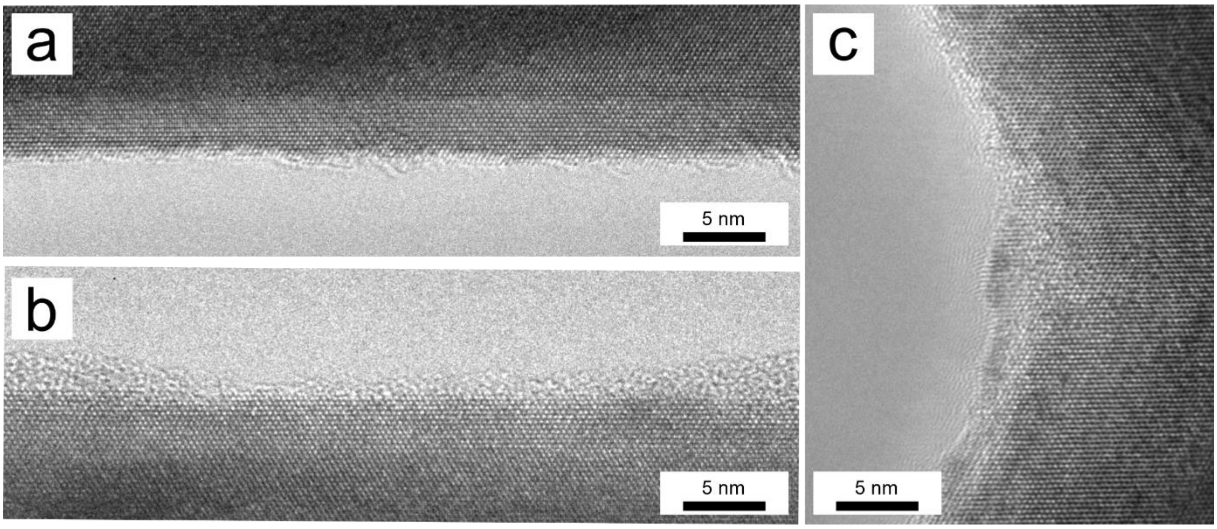

Supplementary Figure 4. (a-c) HRTEM images of two zigzag etched surfaces (a-b) and a corner region (c).

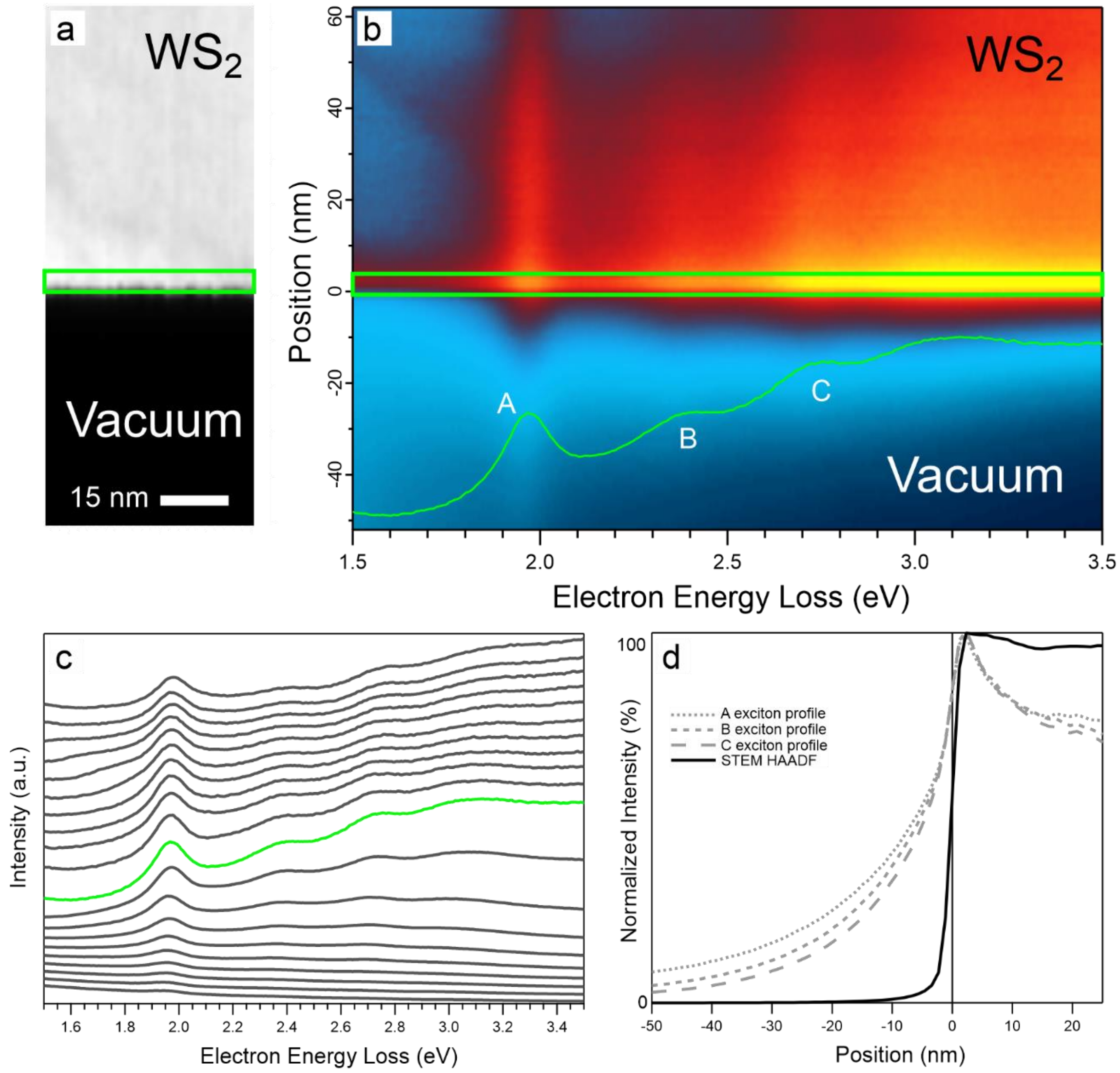

Supplementary Figure 5. (a) HAADF STEM images of the edge of an etched WS_2 sample acquired simultaneously with an EELS spectrum image. (b) EELS spectra extracted from different vertical positions within the spectrum image, starting from the aloof vacuum (bottom) across the WS_2 surface and into the WS_2 (top), and integrated horizontally across the whole spectrum image. All of the spectra in the spectrum image have been normalized to their zero loss peak intensity. The green boxes in (a) and (b) mark the surface region where the green spectra in (b) and (c) were extracted. (c) Spectra extracted from (b) from different vertical positions. The bottom spectrum is from the vacuum, the green spectra is from the surface, and the top spectrum is from the WS_2. The A, B, and C excitons are visible in the spectra for all electron beams positions, including in the vacuum, at the surface, and inside the WS_2. However, the A-, B-, and C-excitons show the largest EELS signal when the electron beam is positioned on the zigzag surface. (d) Spatial profiles of the A-, B-, and C-exciton EELS peak intensities normalized to their max intensity. Negative positions are in the vacuum, 0 nm is the edge of the flake, and positive positions are inside the WS_2. The solid black curve is the normalized ADF STEM intensity, which shows the sharp edge of the WS_2 flake. These profiles demonstrate all excitons are most strongly excitable by EELS at the zigzag surface. Additionally, the A-, B-, and C-excitons are measurable in EELS using an aloof electron beam and the spatial excitation profiles for each are different. For example, the A exciton is excitable in vacuum further away from the WS_2 flake than the B- and C-excitons.

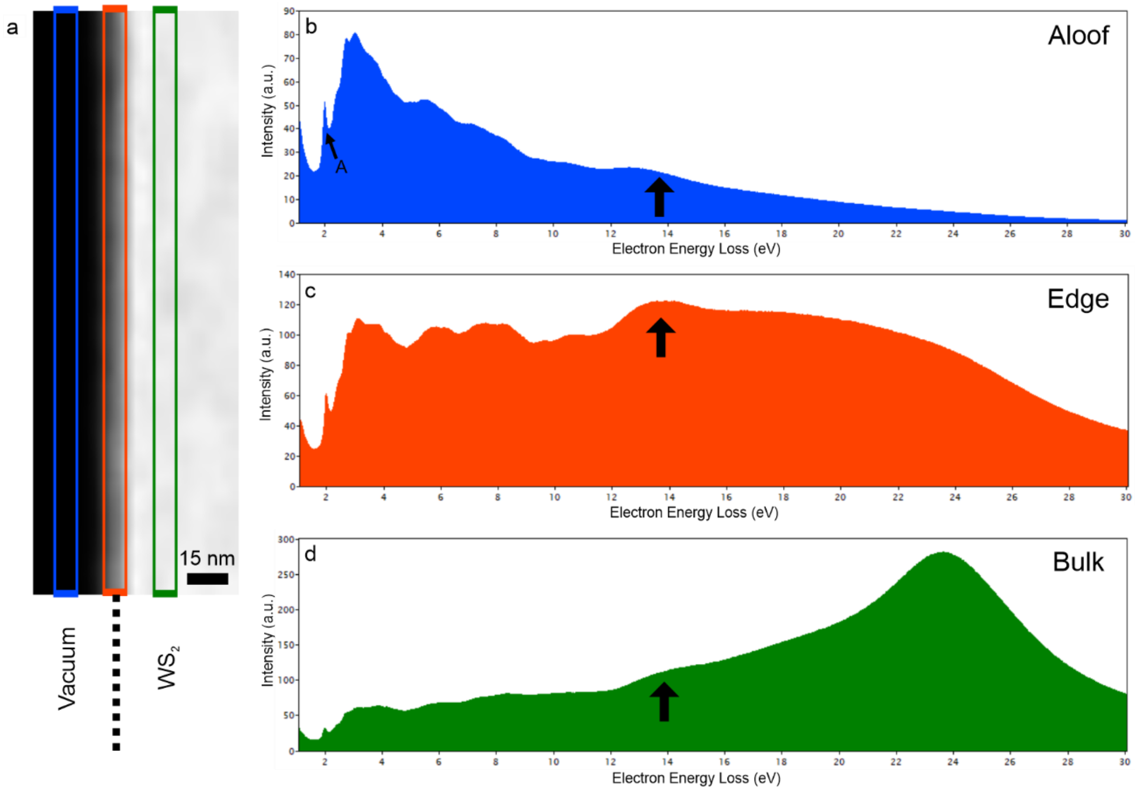

Supplementary Figure 6. (a) HAADF STEM images of the edge of an etched WS_2 sample acquired simultaneously with an EELS spectrum image. On the left is vacuum and on the right is the WS_2 flake. (b-d) EELS spectra extracted from different positions within the spectrum image. (b) The aloof vacuum spectrum extracted from the blue box in (a). (c) The WS_2 edge spectrum extracted from the orange box in (a). (c) The WS_2 bulk spectrum extracted from the green box in (a). The excitons are visible in all spectra, such as the A exciton which is marked in (B) at ~2 eV. The black arrows at ~13.8 eV mark a peak that is present in all spectra but is most prevalent in the spectrum acquired at the edge. This peak is consistent with a peak observed in a previous study of MoS_2 [1], which concluded it is caused by the metallic states of the zigzag edge.

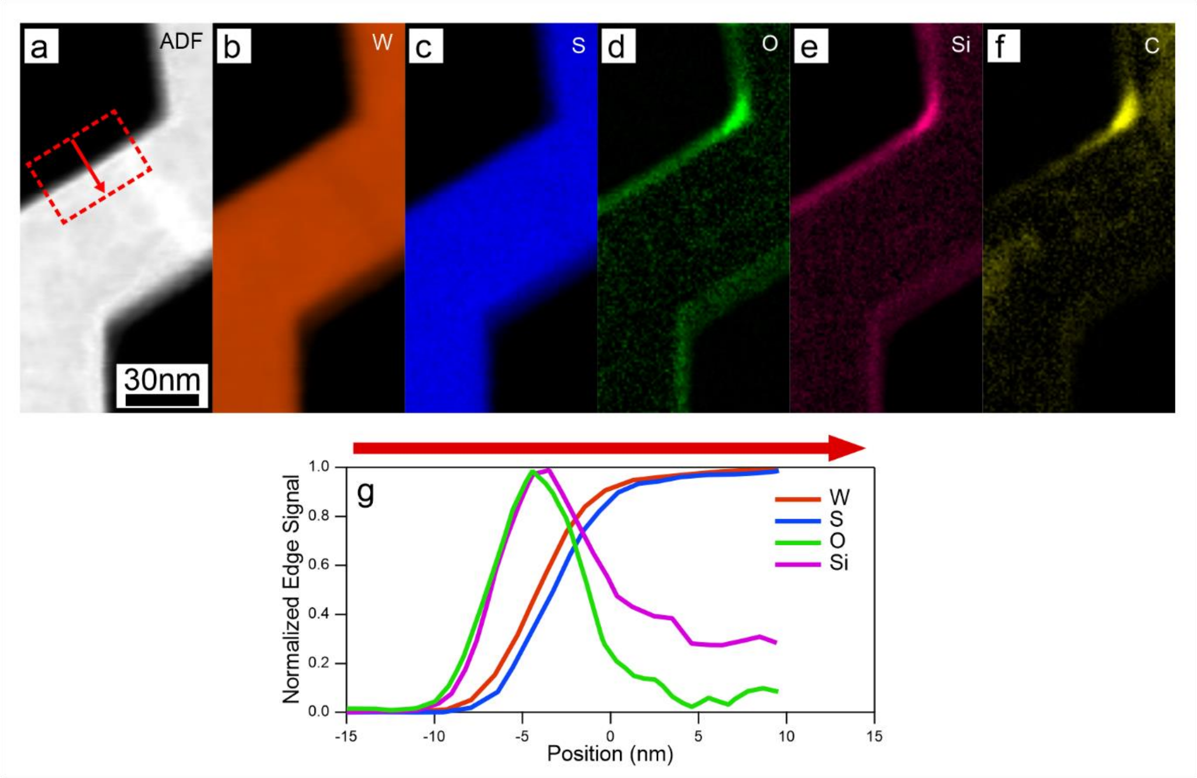

Supplementary Figure 7. (a-f) Core-loss EELS spectrum image data revealing the composition variations of a WS_2 sample etched with a hexagon array pattern. (a) The HAADF STEM image acquired simultaneously with the EELS spectrum image. (b-f) W O, S L, O K, Si L, C K edge signal maps, respectively. (g) W (orange), S (blue), O (green), and Si (pink) EELS signal profile along the red arrow direction in (a) and integrated perpendicular to the arrow within the red boxed marker. Each edge profile has been normalized to its maximum profile signal.

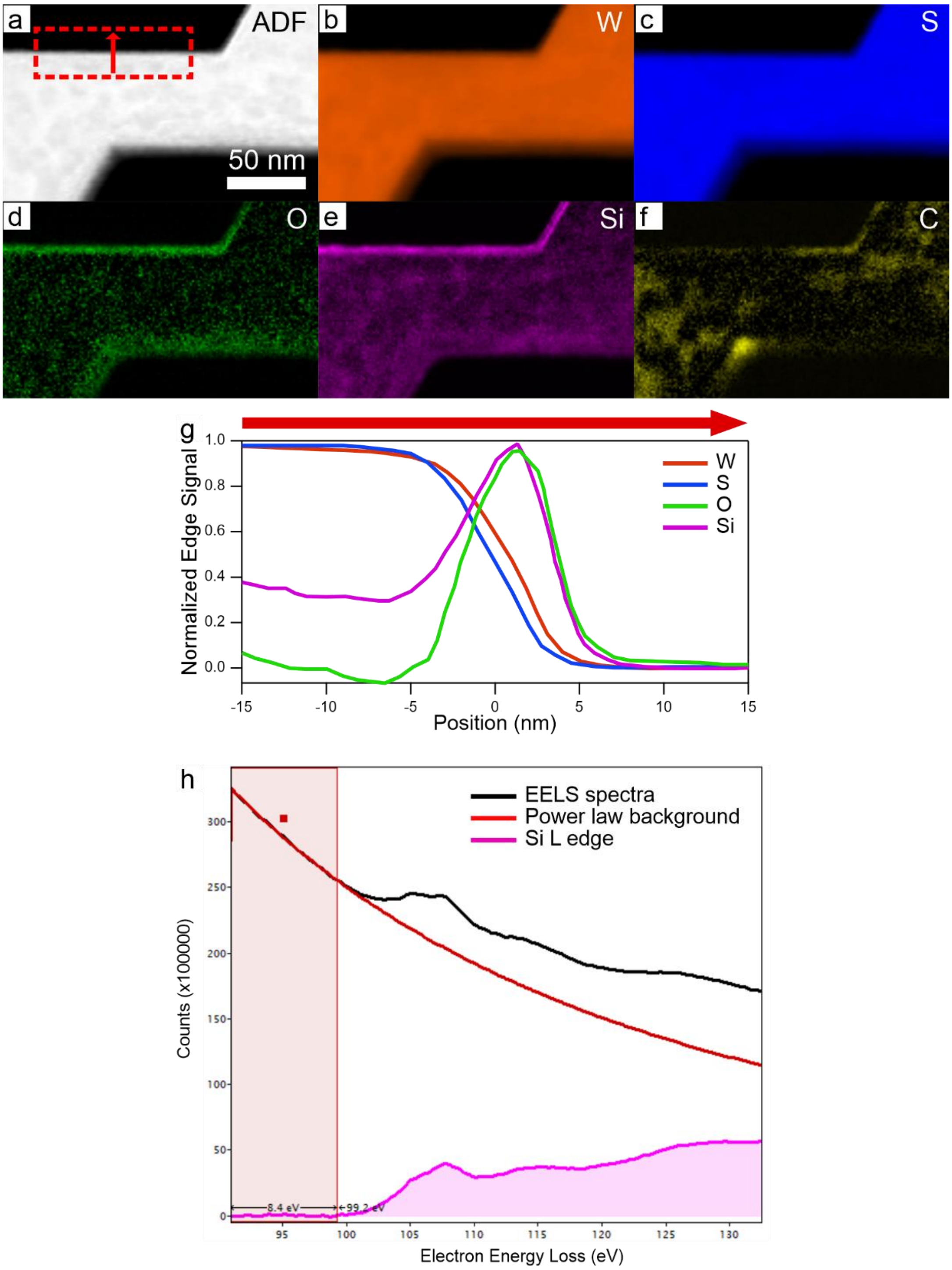

Supplementary Figure 8. EELS. (a-f) Core-loss EELS spectrum image data revealing the composition variations of a WS_2 sample etched with a hexagon array pattern. (a) The HAADF STEM image acquired simultaneously with the EELS spectrum image. (b-f) W O, S L, O K, Si L, C K edge signal maps, respectively. (g) W (orange), S (blue), O (green), and Si (pink) EELS signal profile along the red arrow direction in (a) and integrated perpendicular to the arrow within the red boxed marker. Each edge profile has been normalized to its maximum profile signal. (h) The Si L edge extracted from the spectrum image at the area of high Si intensity at the etched edge, revealing the Si edge characteristics shows more similarities with SiO_2 than pure Si or SiC [2]. The black curve is the raw spectrum. The red curve is the power law background signal determined from the pre-edge region identified by the red box. The pink curve is the background subtracted Si L edge spectrum.

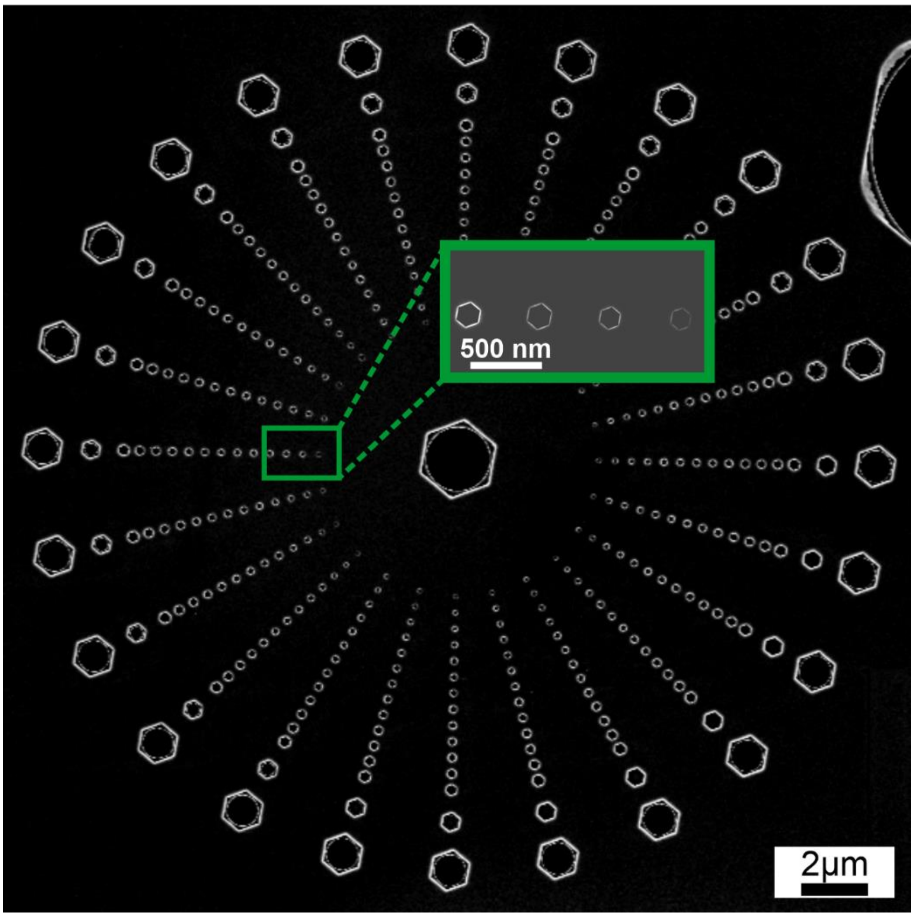

Supplementary Figure 9. SEM image of anisotropically etched single holes in WS_2 flake with various sizes ranging from ~50 nm to ~10 μm in diameter, illustrating the same orientation of individual hexagonal holes.

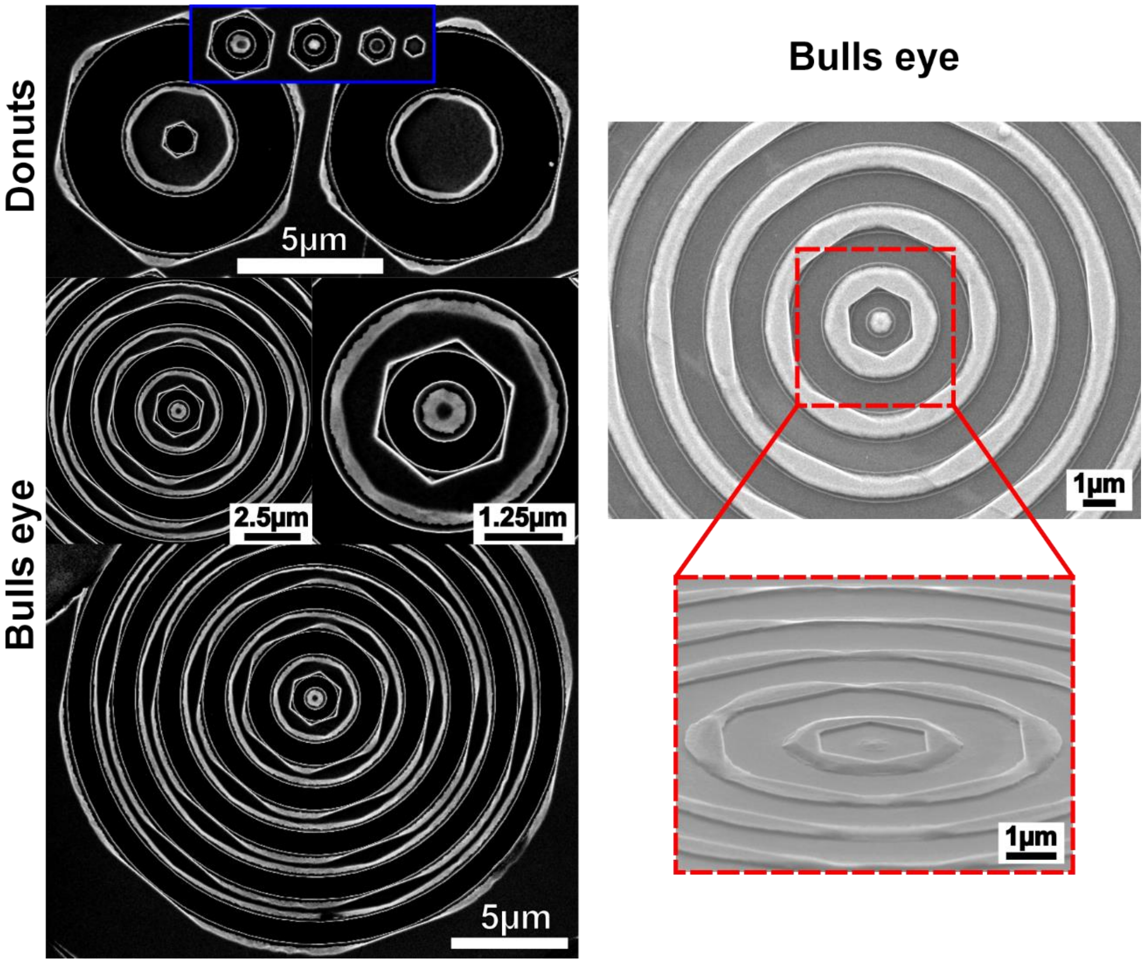

Supplementary Figure 10. SEM images of the donut and bull-eye structures. The inner edges of the etched donuts and bulls-eye structures have well-defined sharp zigzag edges, whereas the outer edges are rotated by 30^0 and have less-distinct armchair character. This suggests that donuts and bulls-eye structures possess alternating metallic zigzag and semiconducting armchair edges.

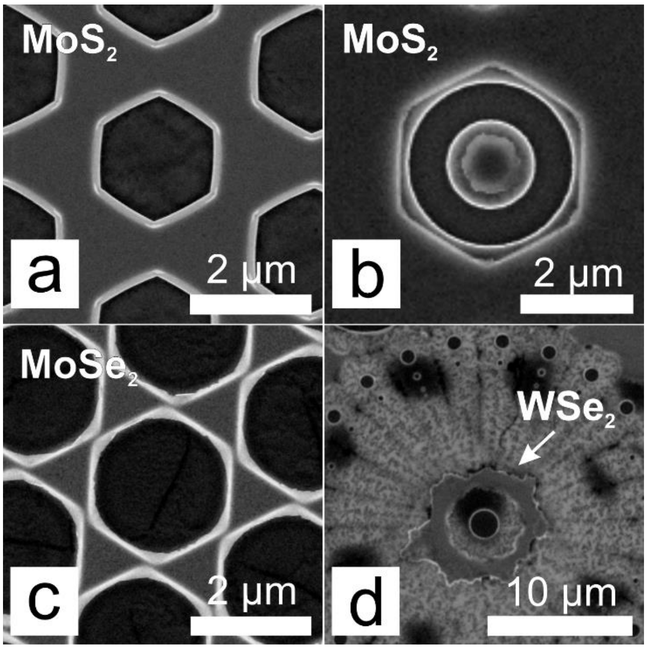

Supplementary Figure 11. SEM images of other TMD materials: (a) high-quality MoS_2 and (b) MoS_2 exfoliated from a natural mineral, (c) $MoSe_2$, and (d) WSe_2 (in this case the etching does not work, possibly due to WSe_2 basal plane being oxidized (see a related work about O_3 treatment of WSe_2 multilayers [3]), we thus conclude that our method is not suitable for etching WSe_2).

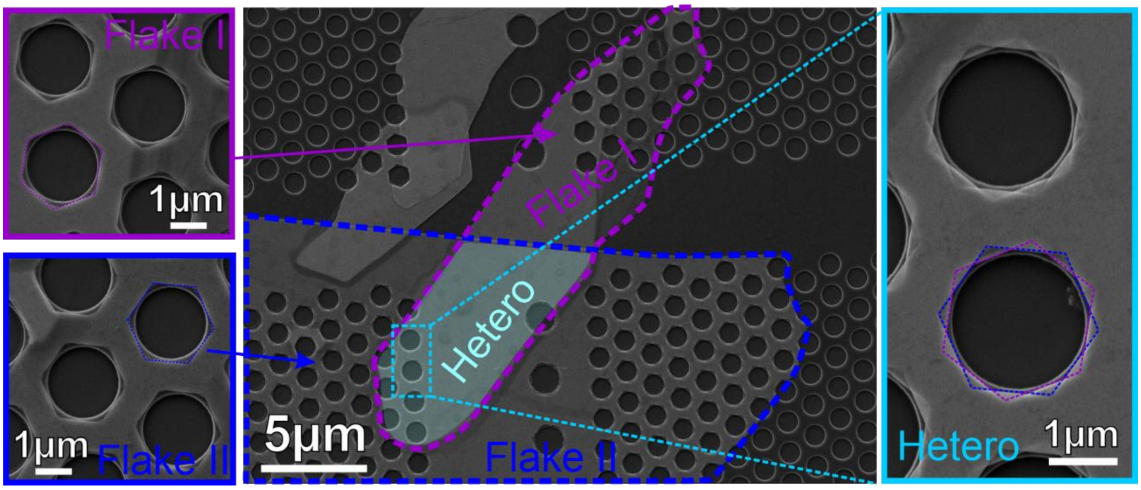

Supplementary Figure 12. Anisotropic etching of a heterostructure composed of WS_2 multilayer flakes rotated respect to their crystallographic axis. The heterostructure was prepared by transferring Flake II on top of the Flake I with a 30^0 rotation between the two, on top of a SiO_2 (55 nm)/Si substrate. This suggest that the method is applicable for a wide range of TMD heterostructures, and possibly for more than 2 layers composed of different TMD materials.

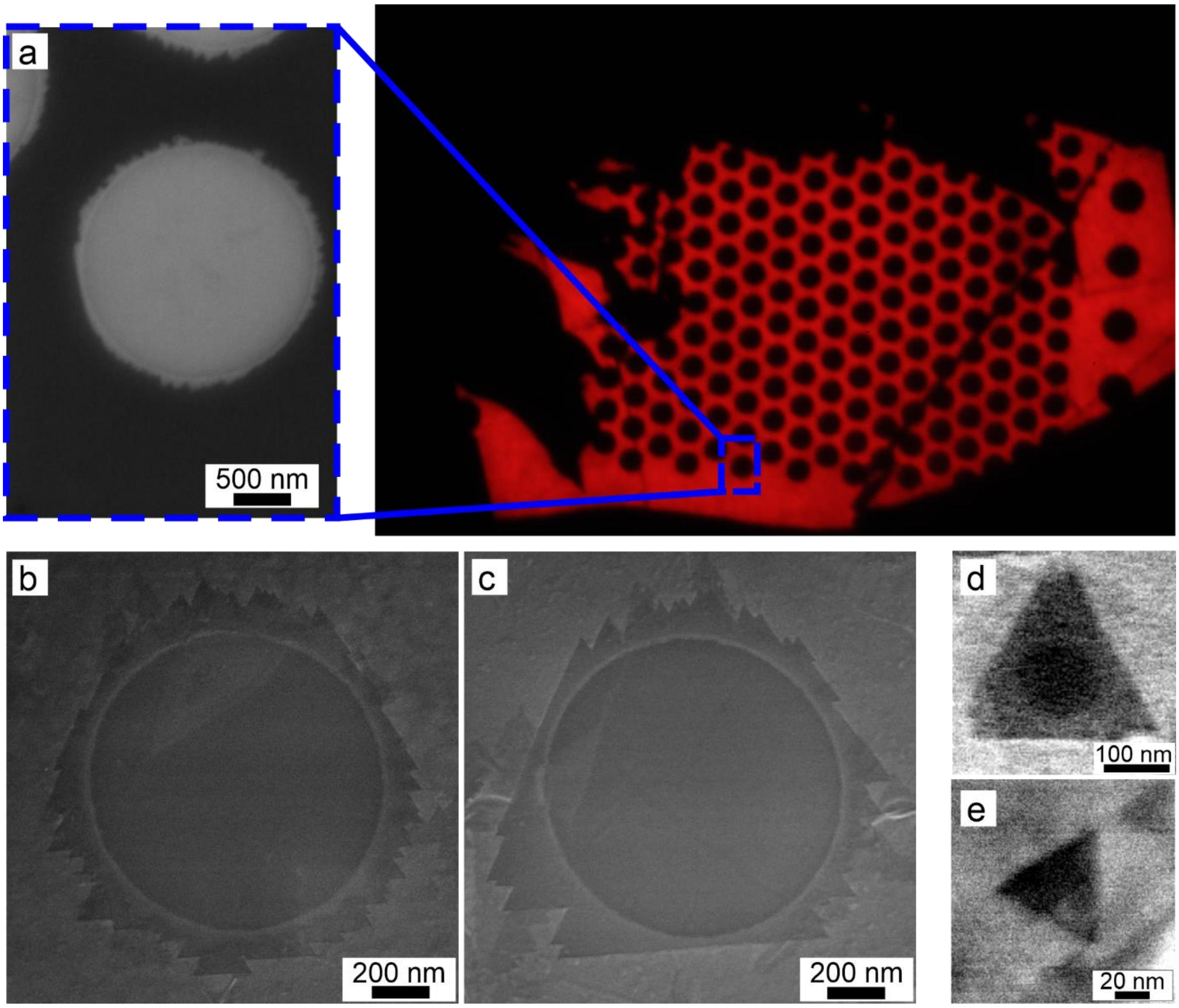

Supplementary Figure 13. (a) SEM (left) and PL (right) images of monolayer WS_2 with 1 µm radius initial holes after ~8 min of anisotropic etching. (b, c) SEM images of heterostructures consisting of two WS_2 monolayers on top each other with 500 nm radius initial holes: (b) after 7 min and (c) 12 min of anisotropic etching, respectively. (d, e) Anisotropically etched triangular holes in WS_2 monolayer with (d) 50 nm and (e) 10 nm radii of initial holes after ~7 min of wet etching.

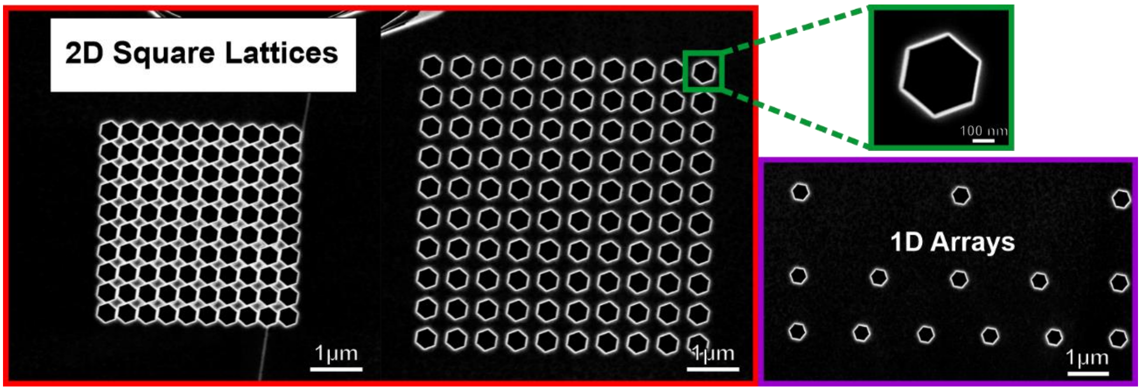

Supplementary Figure 14. SEM images of various square lattice arrays and hole chains in multilayer WS_2.

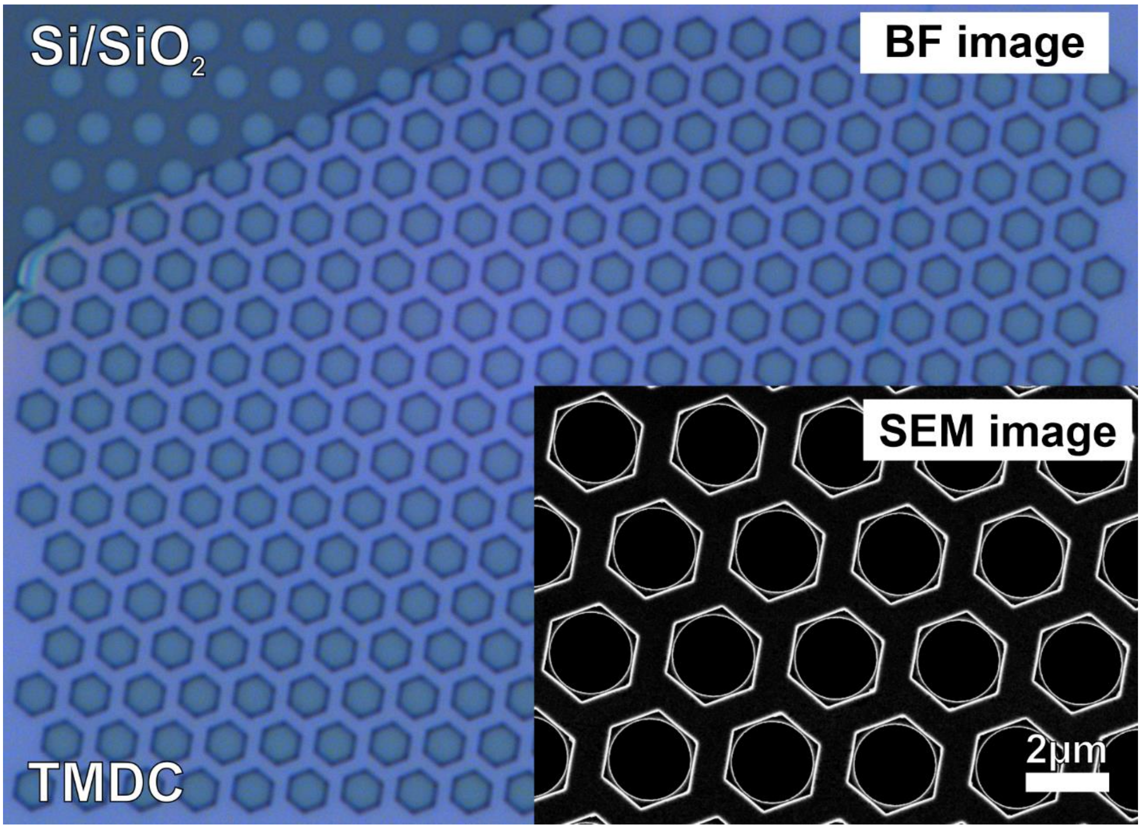

Supplementary Figure 15. Bright-field optical image and SEM image of a hexagonal array with large holes on a SiO_2 (55 nm)/Si substrate. A few tens of nanometer thick WS_2 flake was etched anisotropically using the wet etchant and initial circular holes are transformed into perfect hexagonal holes. On the other hand, the holes in SiO_2 (55 nm)/Si substrate remain circular even after long exposure to the wet etchant.

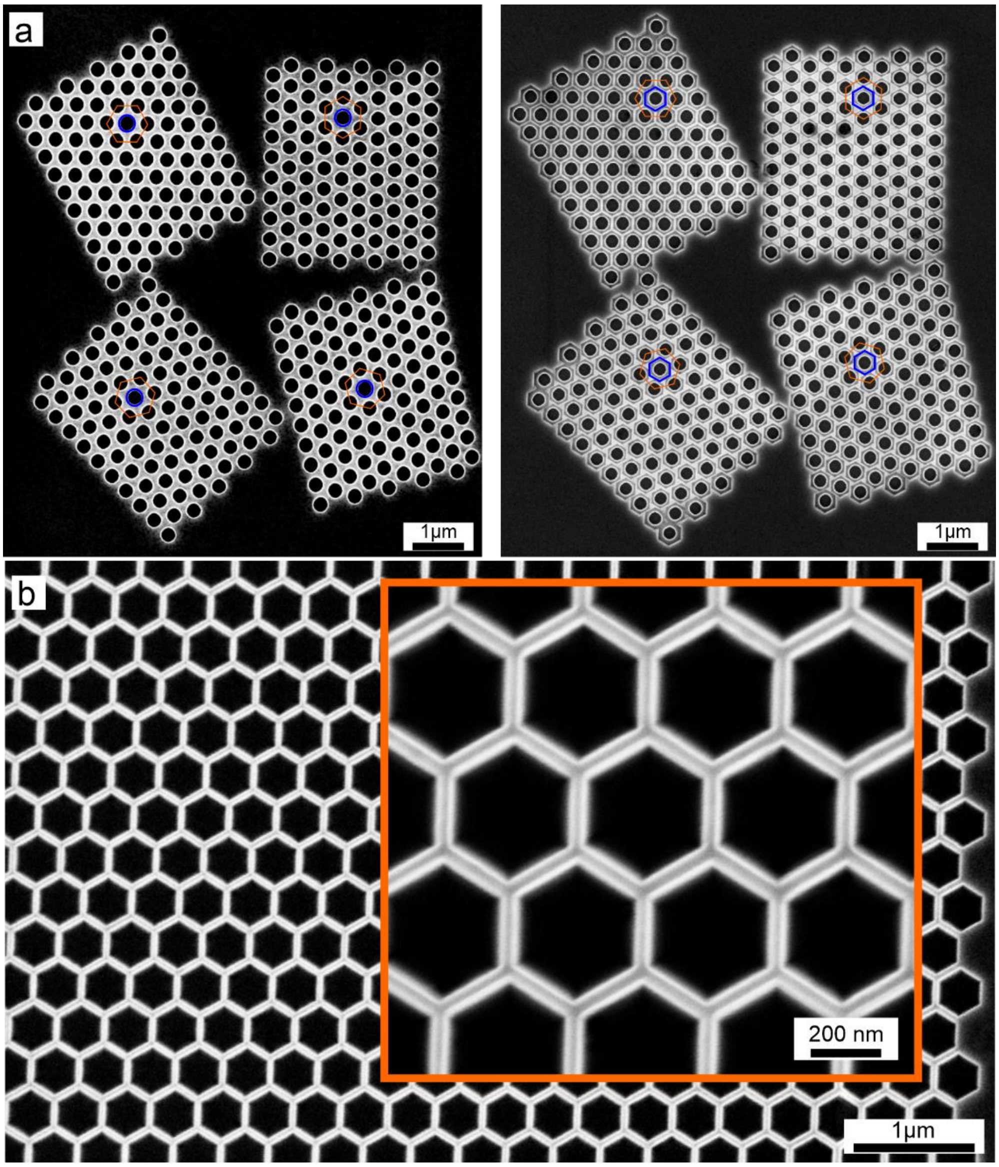

Supplementary Figure 16. (a) SEM images of hexagonal lattice arrays with various rotational angles of the array axis with respect to the crystallographic axis from 0^0 to 45^0 degree with a step of 15^0 degrees. The left image showing the standard substrate with the initial circular holes. The right image is after anisotropic wet etching a WS_2 flake showing the etched hexagonal holes. The orange hexagons and blue hexagons identify the orientations of hexagonal lattice arrays and individual hexagonal holes, respectively. (b) SEM image of an etched WS_2 hexagonal honey-comb array. Inset shows a magnified view of the same array.

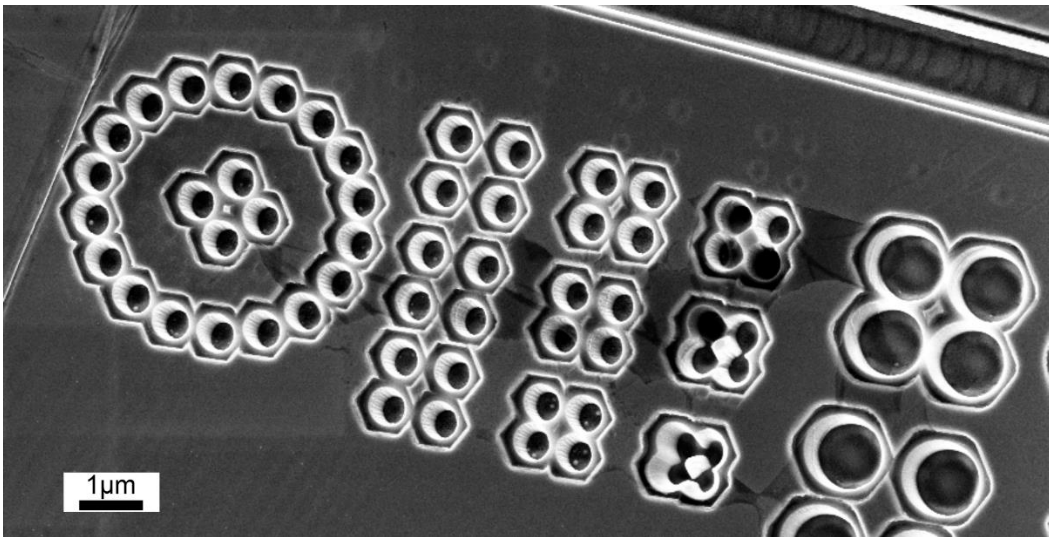

Supplementary Figure 17. SEM image of anisotropically etched hexagonal holes where the initial circular holes were made using focused ion beam (FIB) milling. This technique may be potentially useful from the point of view of maskless-lithography techniques, to prevent potential polymer residue effects on the process. This is mentioned in the generic fabrication scheme in Supplementary Figure 1. The data shows a successful combination of FIB milling with our anisotropic etching method. However, we note that excitonic properties of TMD materials are modified substantially during the FIB session (for which reason this data is not a part of the main text). It could be attributed to Ga^+ ion implantation, amorphous contamination, and degradation due to SEM imaging during the FIB session. These obstacles may be potentially overcome by employing FIB imaging (instead of SEM) as well as by using alternative ions in FIB, for instance, helium. The data shown here indicates that the anisotropic etching method presented here can be readily combined with various types of milling and/or traditional patterning techniques.

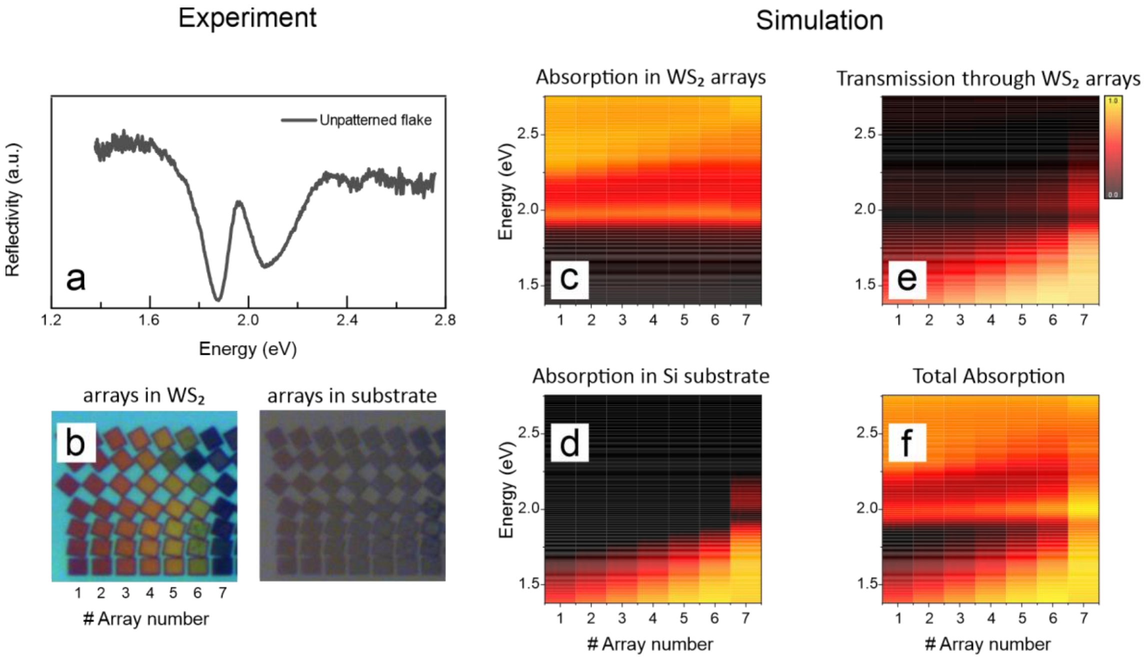

Supplementary Figure 18. (a) Normalized reflectivity spectrum from the unpatterned multilayer WS_2 flake (~70 nm), showing the self-hybridized exciton-polaritons due to strong interaction between excitonic and Fabry-Pérot within the flake [4], leading to a pronounced color. (b) Bright-field true-color optical microscope images of (left) nanopatterned arrays in WS_2 flake and (right) same nanopattern in SiO_2/Si substrate (both photographs are takes under the same illumination/collection conditions). The background color in unpatterned WS_2 flake is blue, while after nanopatterning, array colors vary from blue to red, depending on the array parameters. Note, that similar arrays fabricated in a bare substrate do not produce similar bright colors. (c-e) FDTD simulations of absorption and transmission spectra in/through corresponding WS_2 arrays on SiO_2/Si substrate. (c) Absorption in WS_2 arrays, (d) absorption in underlying Si substrate, (e) transmission through the WS_2 array, (f) total absorption in WS_2 arrays and Si substrate. Note that the total absorption in array #7 is the highest among all arrays, which correspond to its dark color in (b).

Supplementary References:

1 Tinoco, M., Maduro, L. & Conesa-Boj, S. Metallic edge states in zig-zag vertically-oriented MoS_2 nanowalls. *Scientific Reports* **9**, 15602, (2019).

2 Kimoto, K., Sekiguchi, T. & Aoyama, T. Chemical shift mapping of Si L and K edges using spatially resolved EELS and energy–filtering TEM. *Journal of Electron Microscopy* **46**, 369-374, (1997).

3 Yamamoto, M. *et al.* Self-Limiting Layer-by-Layer Oxidation of Atomically Thin WSe_2. *Nano Letters* **15**, 2067-2073, (2015).

4 Munkhbat, B. *et al.* Self-Hybridized Exciton-Polaritons in Multilayers of Transition Metal Dichalcogenides for Efficient Light Absorption. *ACS Photonics*, **6**, 139-147 (2018).
